# IL-1 Signaling in Obesity-Induced Hepatic Lipogenesis and Steatosis

**DOI:** 10.1371/journal.pone.0107265

**Published:** 2014-09-12

**Authors:** Kimberly A. Negrin, Rachel J. Roth Flach, Marina T. DiStefano, Anouch Matevossian, Randall H. Friedline, DaeYoung Jung, Jason K. Kim, Michael P. Czech

**Affiliations:** 1 Program in Molecular Medicine, University of Massachusetts Medical School, Worcester, Massachusetts, United States of America; 2 Department of Medicine, Division of Endocrinology, Metabolism, and Diabetes, University of Massachusetts Medical School, Worcester, Massachusetts, United States of America; University of Hong Kong, China

## Abstract

Non-alcoholic fatty liver disease is prevalent in human obesity and type 2 diabetes, and is characterized by increases in both hepatic triglyceride accumulation (denoted as steatosis) and expression of pro-inflammatory cytokines such as IL-1β. We report here that the development of hepatic steatosis requires IL-1 signaling, which upregulates Fatty acid synthase to promote hepatic lipogenesis. Using clodronate liposomes to selectively deplete liver Kupffer cells in *ob/ob* mice, we observed remarkable amelioration of obesity-induced hepatic steatosis and reductions in liver weight, triglyceride content and lipogenic enzyme expressions. Similar results were obtained with diet-induced obese mice, although visceral adipose tissue macrophage depletion also occurred in response to clodronate liposomes in this model. There were no differences in the food intake, whole body metabolic parameters, serum β-hydroxybutyrate levels or lipid profiles due to clodronate-treatment, but hepatic cytokine gene expressions including IL-1β were decreased. Conversely, treatment of primary mouse hepatocytes with IL-1β significantly increased triglyceride accumulation and Fatty acid synthase expression. Furthermore, the administration of IL-1 receptor antagonist to obese mice markedly reduced obesity-induced steatosis and hepatic lipogenic gene expression. Collectively, our findings suggest that IL-1β signaling upregulates hepatic lipogenesis in obesity, and is essential for the induction of pathogenic hepatic steatosis in obese mice.

## Introduction

The prevalence of obesity represents a fully fledged epidemic as greater than 300 million adults are clinically obese worldwide [1]. Obesity is a prominent risk factor for insulin resistance (IR), type 2 diabetes, non-alcoholic fatty liver disease (NAFLD) and other metabolic disorders because it impairs systemic metabolic homeostasis. A major hallmark of obesity is adipose tissue (AT) dysfunction, which is characterized by a chronic state of low-grade inflammation, and by a decreased ability of adipocytes to efficiently store excess nutrients and lipids as triglycerides (TGs) [1], [2], [3]. This in turn is thought to increase circulating free fatty acids (FFAs) and ectopic lipid deposition within insulin sensitive tissues, such as muscle and liver, causing IR. The intracellular hepatic lipid accumulation and subsequent formation of lipid droplets within hepatocytes can activate resident tissue macrophages, otherwise denoted as Kupffer cells (KCs), which release pro-inflammatory cytokines, including TNF-α, IL-6 and IL-1β [4], [5], [6]. This inflammation enhances NAFLD progression to fibrosis, cirrhosis, chronic liver disease, and exacerbates IR [4], [5], [6], [7], [8].

Recently, macrophage depletion techniques have been used to determine the effects of macrophage function on insulin sensitivity. Conditional macrophage ablation in combination with transgenic and gene deletion mouse models have demonstrated that CD11c^+^ macrophages, Nlrp3-inflammasome components and pro-inflammatory cytokines promote glucose intolerance and IR in both diet-induced obesity (DIO) and genetic mouse models of obesity [9], [10], [11], [12], [13], [14], [15]. Drug-encapsulated liposome administration has been utilized to selectively deplete KCs and visceral adipose tissue macrophages (VATMs) to improve glucose and insulin sensitivity and reduce hepatic steatosis in DIO mouse models [12], [16], [17]. Lastly, anti-cytokine therapy or pharmacological blockade of pro-inflammatory cytokines has also been successful in improving systemic glucose and insulin tolerance as well as β-cell function in obese mice and human subjects [14], [18], [19]. One such drug is Anakinra (Kineret), which is recombinant IL-1Ra that blocks IL-1 signaling via the IL-1 receptor [18], [20], [21]. However, previous work utilizing clodronate liposomes to deplete KCs in DIO mice have reported contradictory conclusions regarding the involvement of KCs in obesity-driven steatosis, clouding the roles of these cells in this syndrome [12], [22], [23], [24].

The present studies were designed to clarify this issue and elucidate the mechanism by which KC-derived pro-inflammatory cytokines regulate whole body and hepatic lipid metabolism in obesity. We targeted KCs *in* *vivo* by intraperitoneal (i.p.) clodronate liposome administration in two animal models of obesity: DIO and *ob/ob* mice. The results herein demonstrate that clodronate liposome-mediated KC depletion, regardless of VATM content in both DIO and *ob/ob* mice, abrogated hepatic steatosis by reducing hepatic *de* *novo* lipogenic gene expression. Additionally, we observed significant decreases in hepatic inflammation and hypothesized that IL-1β may be responsible for the increased TG accumulation in obese mouse livers. In agreement with this hypothesis, IL-1β treatment increased hepatic lipid deposition and Fas expression in primary mouse hepatocytes. Furthermore, the pharmacological inhibition of IL-1 signaling by administration of recombinant human IL-1Ra to DIO mice attenuated obesity-induced hepatic steatosis and reduced hepatic lipogenic gene expression. These data illustrate the importance of IL-1β in obesity-driven hepatic steatosis, and suggest that liver inflammation controls hepatic lipogenesis in obesity.

## Methods

### Animals, Diets and Treatments

Wild type male C57Bl/6J and *ob/ob* mice were purchased from Jackson laboratories (Bar Harbor, ME). Animals were fed ad libitum with free access to water and housed in the University of Massachusetts Medical School (UMASS) Animal Medicine facility with a 12∶12-h light-dark cycle. Animals were weighed weekly for the duration of the diet study. Animals utilized for the clodronate-encapsulated liposome and IL-1Ra studies were fed a high fat diet (HFD, 45 kcal% fat; D12451; Research Diets) starting at 4 wks of age for 13–14 weeks. 8-week-old *ob/ob* mice were fed a standard chow diet (LabDiet PicoLab 5053). Liposome-encapsulated clodronate (250 mg/kg) or an equivalent volume of PBS-liposomes was administered twice i.p. with a three day interval over the course of 6 days in the final week of the HFD-challenge or a week prior to *ob/ob* mouse tissue isolation. Mice were started on daily i.p. injections of recombinant human IL-1 receptor antagonist (IL-1Ra) (32 mg/kg) or saline (Anakinra; Amgen) starting after 9 weeks of HFD and 32 days prior to sacrifice. Intraperitoneal glucose tolerance tests (IPGTTs) were performed as previously described after 28 daily IL-Ra injections [25]. Measurements of energy expenditure, respiratory exchange ratio, indirect calorimetry, and physical activity using metabolic cages (TSE Systems, Bad Homburg, Germany) were performed by the UMass Mouse Metabolic Phenotyping Center. At experimental completions, mice were fasted for 6 hours at the start of the light cycle and then euthanized with CO_2_ inhalation and cervical dislocation. All of the experiments were performed in accordance with protocols approved by the Institutional Animal Care and Use Committees (IACUC) at UMass Medical School.

### Sample Storage

Liver and VAT were harvested and snap frozen in liquid nitrogen for RNA and protein or optimal cutting temperature (OCT) compound for Oil-Red-O analysis. Blood was drawn via the retro-orbital sinus into EDTA tubes; plasma was centrifuged at 10,000 rpm for 10 min and aliquotted. All of the samples were stored at −80°C.

### Primary cell isolation and culture

Anesthetized 8-week old WT mice were perfused via the inferior vena cava as previously described [26]. Briefly, mice were perfused with a 0.5 mM EGTA solution followed by enzymatic collagenase digestion (Sigma) in 1 mM CaCl_2_. Hepatocytes were washed with 1 mM CaCl_2_ (3x) and separated by centrifugation. Primary hepatocytes were seeded in 6-well plates. Before starting stimulation experiments, hepatocytes were rested for 3 hours in M199 media containing 1% Penicillin/Streptomycin, 10% FA-free BSA, 2% FBS, Dexamethasone (100 uM), and Insulin (100 nM) at 37°C and 5% CO_2_. Subsequently, culture media was replaced and cells were maintained in M199 media containing Penicillin/Streptomycin, Dexamethasone and Insulin. Insulin was present in all experimental media to maintain the health of the primary cultures. Cells were left untreated or treated with 10 ng/ml recombinant IL-1β (Millipore) for 24 hours.

### RNA isolation and quantitative RT-PCR

Liver and VAT were isolated, snap frozen in liquid nitrogen, and stored at −80°C. Tissues were homogenized using the gentleMACs Dissociator (Miltenyi Biotec) and isolated following the manufacturers protocol (TriPure, Roche). Precipitated RNA was treated with DNAse (DNA-free, Life Technologies) prior to reverse transcription (iScript Reverse transcriptase, BioRad). SYBR green quantitative PCR (iQ SYBR green supermix, BioRad) was performed on the BioRad CFX97. Gene expression was normalized to the ribosomal gene *36B4* and expressed relative to the expression of PBS liposome- or saline-treated mice. The internal loading control, 36B4, did not change with liposome or IL-1Ra treatment. Melt curve analysis was performed to determine the PCR reaction product specificity. Primer sequences were designed with Primer Bank. Primer sequences are as follows: 36B4: 5′-TCCAGGCTTTGGGCATCA-3′, 3′-CTTTATCAGCTGCACATCACTCAGA-5′, CD11c: 5′-CTGGATAGCCTTTCTTCTGCTG-3′,3′-GCACACTGTGTCCGAACTCA-5′; F4/80: 5′-CCCCAGTGTCCTTACAGAGTG-3′, 3′-GTGCCCAGAGTGGATGTCT-5′; IL1β: 5′-GCAACTGTTCCTGAACTCAACT-3′, 3′-ATCTTTTGGGGTCCGTCAACT-5′; TNFα: 5′-CAGGCGGTGCCTATGTCTC-3′, 3′-CGATCACCCCGAAGTTCAGTAG-5′; IL-6: 5′-TAGTCCTTCCTACCCCAATTTCC-3′, 3′-TTGGTCCTTAGCCACTCCTTC-5′; IL-1α: 5′-GCACCTTACACCTACCAGAGT-3′, 3′- TGCAGGTCATTTAACCAAGTGG-5′; NLRP3: 5′-ATTACCCGCCCGAGAAAGG-3′, 5′-TCGCAGCAAAGATCCACACAG-3′, FASn: 5′-GGAGGTGGTGATAGCCGGTAT-3′, 5′-TGGGTAATCCATAGAGCCCAG-3′, DGAT: 5′-TCCGTCCAGGGTGGTAGT-3′, 5′-TGAACAAAGAATCTTGCAGACGA-3′, ELOV6: 5′-GAAAAGCAGTTCAACGAGAACG-3′, 5′-AGATGCCGACCACCAAAGATA-3′, ACC2: 5′-GGAGGCTGCATTGAACACAAGT-3′, 5′-TGCCTCCAAAGCGAGTGACAAA-3′, PPARG2: 5′- ATGGGTGAAACTCTGGGAG-3′, 5′-GTGGTCTTCCATCACGGAGA-3′, SCD1: 5′-TTCTTGCGATACACTCTGGTGC-3′, 5′-CGGGATTGAATGTTCTTGTCGT-3′, PPARα: 5′-AGAGCCCCATCTGTCCTCTC-3′, 5′-ACTGGTAGTCTGCAAAACCAAA-3′, CPT1A: 5′-GCTGCTTCCCCTCACAAGTTCC-3′, 5′-GCTTTGGCTGCCTGTGTCAGTATGC-3′, BAX: 5′-AGACAGGGGCCTTTTTGCTAC-3′, 5′-AATTCGCCGGAGACACTCG-3′, BAK: 5′-CAGCTTGCTCTCATCGGAGAT-3′, 5′-GGTGAAGAGTTCGTAGGCATTC-3′.

### Protein Analysis

Tissue pieces were homogenized in a Dounce homogenizer in RIPA protein lysis buffer [50 mM Tris (pH 7.4), 0.1% SDS, 400 mM NaCl, 0.5% deoxycholate, 1% NP40,1 mM EDTA, 25 mM sodium fluoride, 1 mM sodium orthovanadate, 1 mM benzamidine, 1 mM phenylmethylsulfonyl fluoride, and 10 µg/ml of aprotinin and leupeptin. Primary hepatocytes were homogenized in lysis buffer (150 mM NaCl, 2% SDS, and 2 mM EDTA, 25 mM sodium fluoride, 1 mM sodium orthovanadate, 1 mM benzamidine, 1 mM phenylmethylsulfonyl fluoride, and 10 µg/ml of aprotinin and leupeptin. Samples were sonicated using a microtip and protein content was quantified using a BCA protein assay kit (Thermo Scientific). Proteins were resolved on a 10% SDS-PAGE gel, transferred to a nitrocellulose membrane, blocked with 5% non-fat milk in TBST (0.05% Tween 20 in Tris-buffered saline), washed with TBST, and incubated with primary antibodies overnight. The blots were washed with TBST, and a horseradish peroxidase secondary antibody was applied. Proteins were visualized using Western Lightening Plus ECL (PerkinElmer). Primary antibodies used were Fas and β-Actin (Cell Signaling; 1∶1000 and 1∶10,000).

### Histology

Livers were isolated and fixed in 10% formalin, paraffin embedded, and stained with hematoxylin and eosin (H&E) or frozen in OCT and stained with Oil-Red-O. Images were taken with an Axiovert 35 Zeiss microscope (Zeiss, Germany) equipped with an Axiocam CCl camera at 10x or 20x magnification.

### Non-Esterified Fatty Acid (NEFA) measurements

EDTA plasma was collected from the retro-orbital sinus after isoflurane anesthesia. NEFA were measured with a colorimetric assay (WAKO) using the manual procedure according to manufacturer’s instructions.

### Triglyceride measurements

Total hepatic TG content measurement was performed as previously described [27]. Briefly, total lipids were extracted from liver samples (100 mg) or primary hepatocyte cultures using a 2∶1 mixture of chloroform and methanol. The organic layer was dried overnight and reconstituted in a solution containing 60% butanol and 40% of a 2∶1 mixture of Triton-X114 and methanol. Total TGs were measured with a colorimetric assay (Sigma) using the manual procedure according to manufacturer’s instructions.

### ELISA Assay

EDTA plasma was collected from the retro-orbital sinus after isoflurane anesthesia. The exogenously administered IL-1Ra was measured using specific ELISA recognizing human IL-1Ra (R&D systems Inc.) and fasting insulin levels using a rat/mouse insulin ELISA (Millipore). Both assays were measured in duplicate and according to the manufacturers′ instructions.

### Flow cytometry

The VAT stromal vascular fraction (SVF) was isolated by digestion in HBSS, 2.5% BSA and 2 mg/mL collagenase for 45 minutes and strained through a 70 µm filter followed by red blood cell lysis. Cells were blocked with mouse IgG in FACS buffer (1% BSA/PBS). Cells were stained with antibodies directed towards F4/80 (APC, ABD serotec), CD11b (Percp 5.5, BD), Siglec F (PE, BD) and CD11c (V450, BD). The data were collected on an LSRII flow cytometer (BD) and were analyzed with FlowJo software. Samples were gated for scatter and single cells. Gates were drawn based on fluorescence minus one (FMO) controls. A total of 100,000 events were recorded.

### Liposome Preparation

Cholesterol (16 mg) and phosphatidylcholine (172 mg) (Sigma-Aldrich) were dissolved in chloroform in a round-bottom flask. The chloroform was evaporated at 37°C in a rotary evaporator under vacuum until a thin lipid film formed. 2 g of dichloromethylenediphosphonic acid disodium salt (clodronate) (Sigma-Aldrich) were dissolved in 10 ml of PBS. The clodronate-PBS solution or the control-PBS solution was added to the lipid film and shaken at 4 *g* for 30 minutes. The solution was sonicated for 2 minutes at room temperature in a water bath sonicator (150 watts). The liposomes were washed and centrifuged at 20,000 *g* for 2 hours, and resuspended in 8 ml of PBS. An aliquot of each liposome preparation was subjected to 1∶2 phenol:chloroform extraction to determine the incorporated clodronate concentration within the liposomes and analyzed via liquid chromatography mass spectroscopy on a Waters Acquity UPLC with a Phenomenex 2.1×100 mm Synergi 4u Polar-RP 80A column. A standard curve was created using QuanLynx and samples were then quantified against this calibration curve.

### Statistical Analysis

All of the values are presented as the mean ± SEM. For all experiments a student’s t-test for two-tailed distributions with equal variances was used for comparison between 2 groups. Differences less than p<0.05 were considered to be significant. The area under the curve (AUC) for the IPGTT was calculated using a student’s t-test after performing a baseline correction for the basal glucose values for each individual mouse. All of the data were entered into Microsoft Excel, and statistical analyses were performed with Graph Pad Prism 6.0.

## Results

### Clodronate liposome-mediated KC depletion ameliorates hepatic steatosis in both DIO and *ob/ob* mice

**Figure 1:**
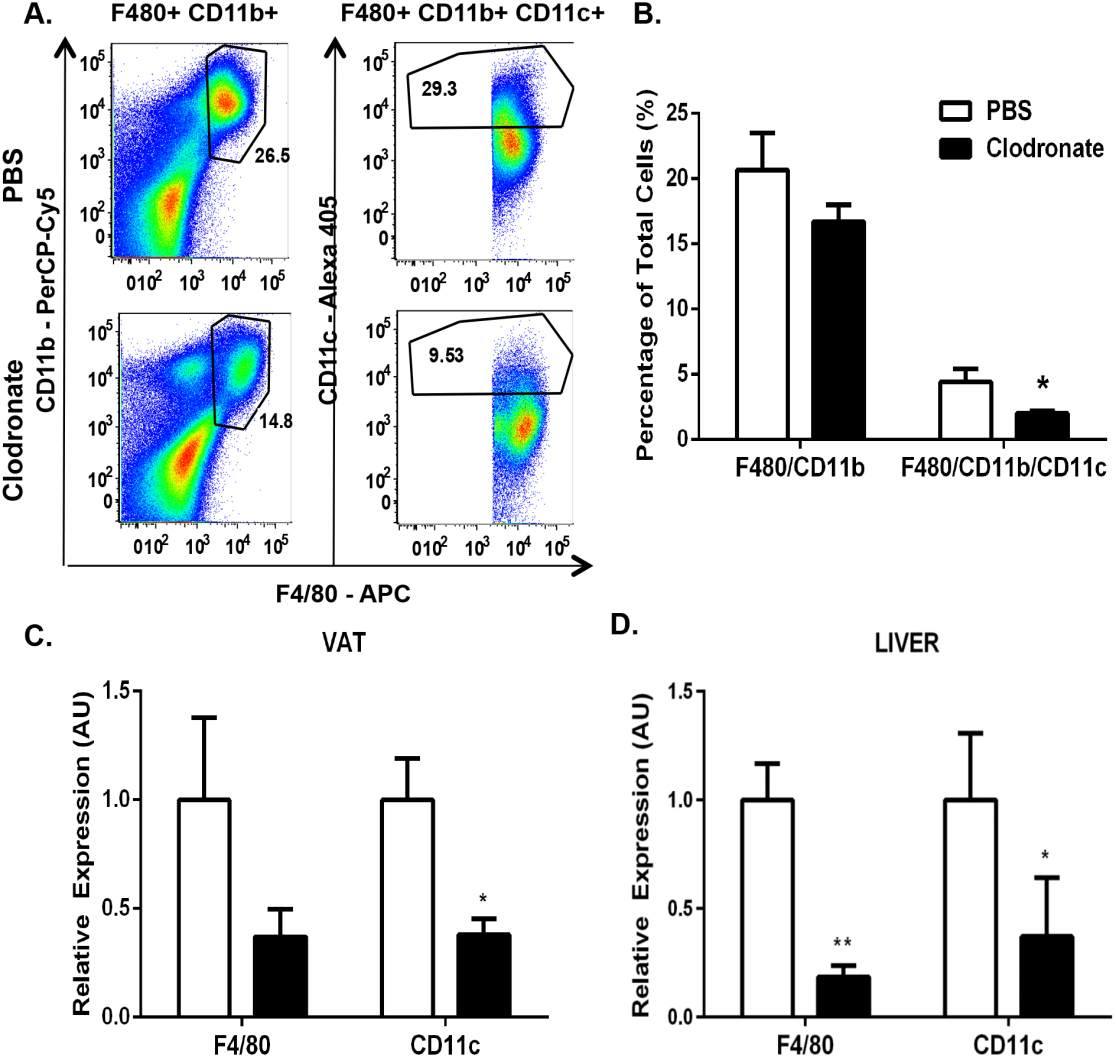
Clodronate liposomes effectively deplete KCs and VATMs of DIO mice. Wild type mice were fed a HFD for 13 weeks and injected with 2 doses of clodronate liposomes (250 mg/kg) or PBS liposomes i.p. over 6 days with a 3-day interval. We observed no changes in the total animal body weight of PBS liposome-treated mice (42.4±1.8 to 41.6±1.9) or clodronate-treated mice (44.01±2.2 to 41.5±2.6). VAT SVF was isolated and FACS analysis was performed. *A*.) Upper panels, PBS liposome-treated mice. Lower panels, clodronate-treated mice. Left panels, SiglecF-negative, CD11b- and F4/80-positive cells. Right panels, CD11c-positive cells. Panels are representative of 6 animals per group. *B*.) Percentage of the total number of cells counted for both CD11b- and F4/80-positive cells and CD11c-positive cells in the VAT of DIO mice. RNA was extracted and quantitative RT-PCR was performed for standard macrophage markers (F480, CD11c) in *C.*) VAT and *D*.) liver of DIO mice. Values represent the mean ± SEM. An unpaired student’s t*-*test was used for comparisons between groups. *P≤0.05, **P≤0.01.

Resident tissue macrophages aid in tissue homeostasis, but obesity can promote low-grade inflammation in insulin sensitive tissues. Conflicting literature has complicated our understanding of the role of KCs and hepatic inflammation in lipid homeostasis in obese mouse livers [12], [22], [24], [28]. Thus, we sought to investigate the mechanism whereby KCs and KC-derived cytokines regulate whole body and hepatic lipid metabolism in two mouse models of obesity after clodronate liposome-mediated KC depletion. We employed a 2-dose injection scheme of clodronate liposomes (250 mg/kg) or an equivalent volume of control PBS liposomes to deplete KCs *in* *vivo*. Liposomes were injected i.p. into DIO or *ob/ob* mice every 3-days over the course of 6 days during the final week of a 13-week HFD challenge. It has been established that short-term diet-induced IR is caused by lipotoxicity, which occurs after only 3 days of high fat feeding [29]. Chronic inflammation emerges as a more dominant mechanism once obesity is established by a long-term, 10-week high fat feeding [29]. Therefore, late phase injections were utilized in this study. We observed no changes in the total animal body weight of PBS or clodronate liposome-treated mice upon completion of the experiments (Fig. 1).

Previous studies employing i.p. clodronate liposome administration in DIO mice report inconsistencies in the ability to deplete both KCs and VATMs from the liver and VAT, respectively [12], [24], [28]. To first confirm the efficiency of macrophage depletion within the VAT of the clodronate-treated DIO mice, we isolated the VAT stromal vascular fraction (SVF) on day 6 from animals that had been injected with clodronate or an equivalent volume of PBS liposomes. Cells were characterized by flow cytometry analysis and macrophages were identified as cells that were positive for CD11b and F4/80 antigen and negative for the eosinophil marker Siglec F [30], [31]. In PBS liposome-treated DIO animals, approximately 27% of Siglec F-negative SVF cells were positive for both CD11b and F4/80 (Fig. 1*A*–*B*). However, in clodronate-treated DIO animals, this percentage decreased to 14%, which was a 45% decrease compared with PBS liposome-treated mice. Moreover, the percentage of the M1-type inflammatory macrophage marker, CD11c, of the total SVF cells was reduced 68% in clodronate-treated mice compared with PBS liposome-treated mice (Fig. 1*A*–*B*; p<0.05). As assessed by qRT-PCR, we observed an 82% and 65% decrease in F4/80 and CD11c gene expression, respectively, in clodronate-treated DIO mouse livers compared with control animals (Fig. 1*D*; p≤0.01, 0.05). We also isolated subcutaneous AT (SAT), lung and spleen to determine if clodronate-liposomes are capable of depleting resident tissue macrophages within other tissues. We observed a reduction in splenic macrophage content without affecting macrophages present within the lungs or SAT of clodronate-treated mice compared with PBS liposome-treated control mice (data not shown). We also isolated SAT, lung and spleen to determine if clodronate-liposomes are capable of depleting resident tissue macrophages within other tissues. We also isolated primary hepatocytes from clodronate-treated DIO mice via perfusion and observe no changes in pro-apoptotic gene expression, indicating that the hepatocellular apoptosis pathway is unaffected by clodronate liposome treatment (data not shown). Taken together, these results indicate that both macrophages in VAT and KCs in the liver of DIO mice were greatly reduced by the clodronate treatments.

**Figure 2:**
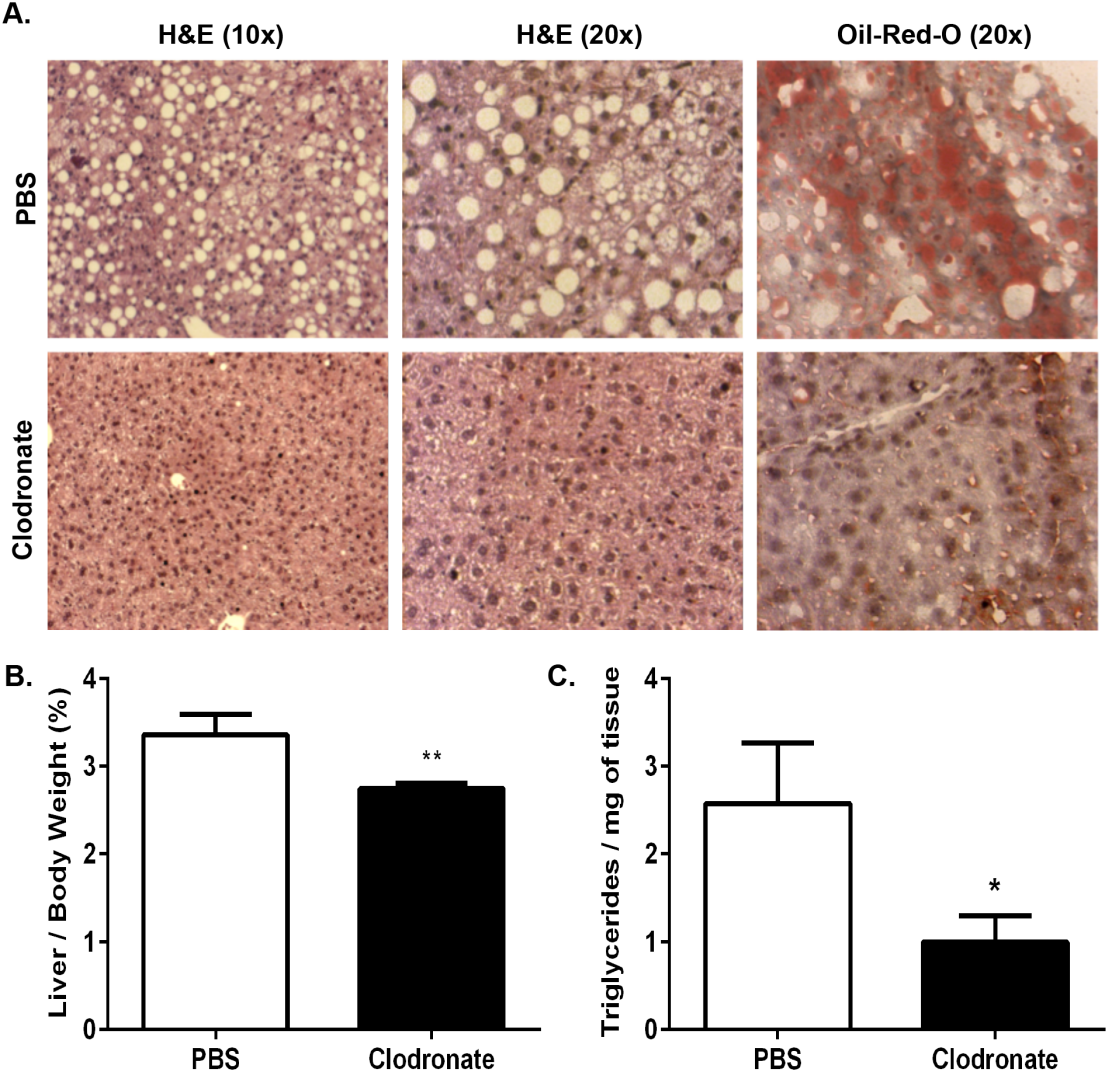
Clodronate liposome-mediated KC and VATM depletion ameliorates hepatic steatosis in DIO mice. Wild type mice were fed a HFD for 13 weeks and injected with 2 doses of clodronate liposomes (250 mg/kg) or PBS liposomes by i.p. over 6 days with a 3-day interval. *A*.) Livers of DIO mice were isolated, fixed in 10% formalin, embedded in paraffin, and stained with H&E or frozen in OCT and stained with Oil-Red-O to assess steatosis. Images are representative of 12–16 animals at 10X and 20X magnification. *B*.) Livers of DIO animals were weighed and data is represented as a percentage of total body weight. *C*.) Total TGs were extracted and normalized to tissue weight. Values represent the mean ± SEM. An unpaired student’s t*-*test was used for comparisons between groups. *P≤0.05, **P≤0.01.

Upon dissection of the mice, we noted a macroscopic reduction in the steatotic appearance of the livers of clodronate-treated DIO animals compared with PBS liposome-treated control mice. In agreement with this observation, DIO mouse liver weights as a percentage of total animal body weight were decreased by 19% in clodronate-treated mice compared with PBS liposome-treated mice (Fig. 2*B*; p≤0.01). Clodronate-treated DIO mice displayed a significant reduction of liver TGs when compared with PBS liposome-treated mice, as observed microscopically by H&E and Oil-Red-O staining (Fig. 2*A*). Quantitative analysis revealed that KC depletion resulted in a 62% decrease in hepatic TG content in clodronate-treated DIO mice compared with the PBS liposome-treated controls (Fig. 2*C*; p≤0.05). These data demonstrate that clodronate-mediated KC and VATM depletion results in a remarkable reduction in hepatic TG content and amelioration of obesity-induced steatosis in DIO mice.

**Figure 3:**
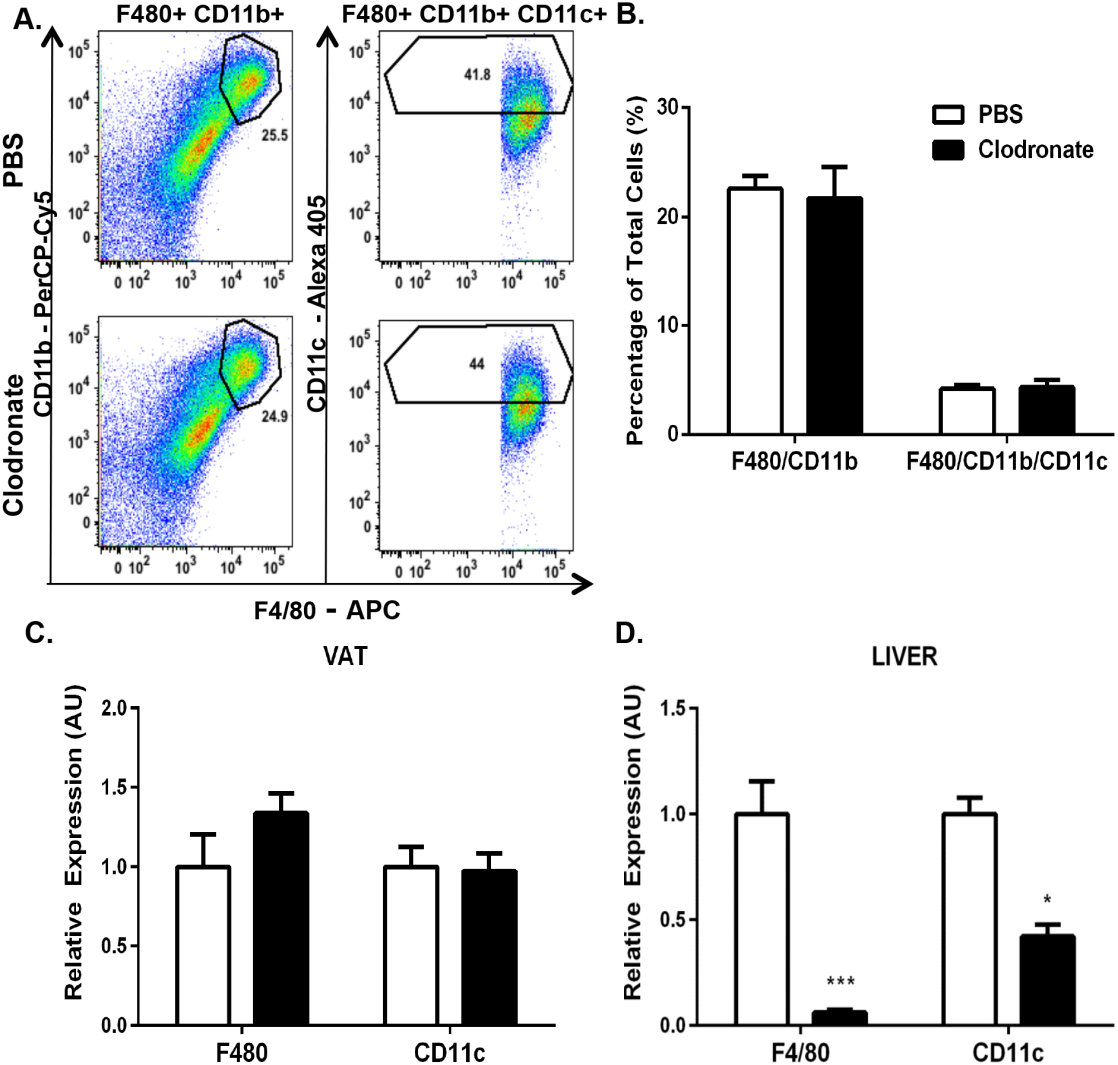
Clodronate-encapsulated liposomes deplete KCs but not VATMs in *ob/ob* mice. 8-week *ob/ob* mice were injected with 2 doses of clodronate liposomes (250 mg/kg) or PBS liposomes by i.p. for 6 days with a 3-day interval. We observed no changes in the total animal body weight of PBS liposome-treated mice (44.2±0.7 to 45.2±0.6) or clodronate -treated mice (41.9±1.4 to 41.1±1.7). VAT SVF was isolated and FACS analysis was performed. *A*.) Upper panels, PBS liposome-treated mice. Lower panels, clodronate-treated animals. Left panels, SiglecF-negative, CD11b- and F4/80-positive cells. Right panels, CD11c-positive cells. Panels are representative of 4–6 animals per group. *B*.) Percentage of the total number of cells counted for both CD11b- and F4/80-positive cells and CD11c-positive cells in the VAT of *ob/ob* mice. RNA was extracted and quantitative RT-PCR was performed for standard macrophage markers (F480, CD11c) in *C*.) VAT and *D*.) liver of *ob/ob* mice. Values represent the mean ± SEM. An unpaired student’s t*-*test was used for comparisons between groups. *P≤0.05, ***P≤0.001.

In order to determine whether these results would also apply to a genetic model of obesity, we used the leptin-deficient *ob/ob* mouse in a subsequent set of experiments. Unexpectedly, no reduction in *ob/ob* mouse VATM content was observed after clodronate treatment as assessed by either FACS analysis or RT-PCR (Fig. 3*A*–*C*). Thus, clodronate treatment depleted VATMs in the DIO mouse model of obesity, but not in *ob/ob* mice when compared with their respective PBS liposome-treated controls. However, we observed a 94% and 60% decrease in F4/80 and CD11c gene expression, respectively, in *ob/ob* mouse livers after clodronate treatment compared with PBS liposome-treated mice (Fig. 3*D*; p≤0.001, 0.05). These data illustrate that clodronate administration can effectively and efficiently deplete KCs from the livers of both DIO and *ob/ob* mice, whereas VATM depletion only occurs in the DIO mouse model.

**Figure 4:**
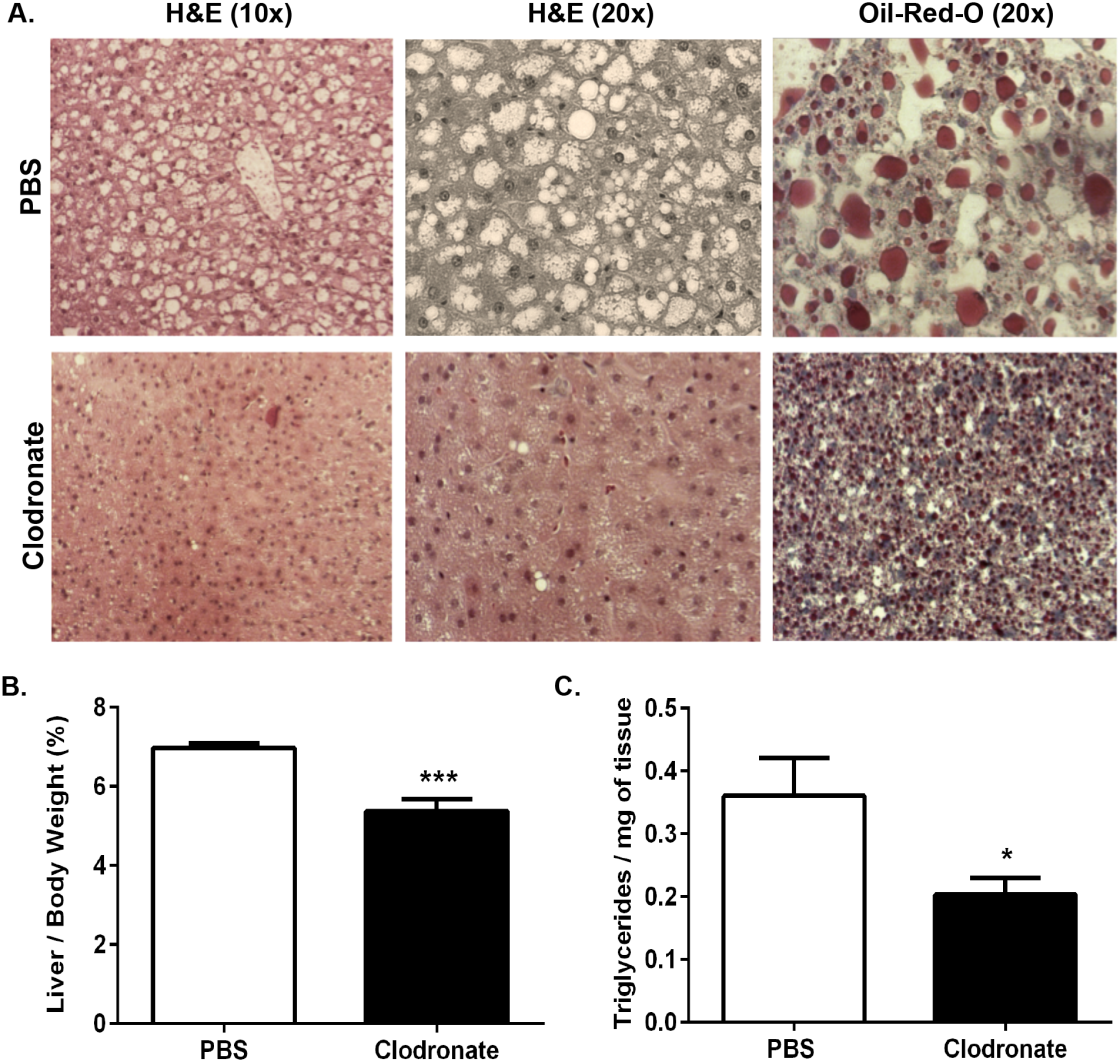
Clodronate liposome-mediated KC depletion ameliorates hepatic steatosis in *ob/ob* mice. 8-week old male *ob/ob* mice were injected with 2 doses of clodronate liposomes (250 mg/kg) or PBS liposomes by i.p. over 6 days with a 3-day interval. *A*.) Livers of *ob/ob* animals were isolated, fixed in 10% formalin, embedded in paraffin, and stained with H&E or frozen in OCT and stained with Oil-Red-O to assess steatosis. Images are representative of 7–10 animals at 10X and 20X magnification. *B*.) Livers of *ob/ob* animals were weighed and data is represented as a percentage of total body weight. *C*.) Total TGs were extracted and normalized to tissue weight. Values represent the mean ± SEM. An unpaired student’s t*-*test was used for comparisons between groups. *P≤0.05, ***P≤0.001.

In spite of the fact that cell depletion due to clodronate was restricted to KCs and not VATMs in the *ob/ob* mouse model, a similar reduction in the steatotic appearance of the livers of clodronate-treated *ob/ob* animals compared with PBS liposome-treated animals was apparent (Fig. 4). In agreement with this observation, *ob/ob* mouse liver weights as a percentage of total body weight were decreased 30% in the clodronate-treated animals compared with PBS liposome-treated animals (Fig. 4*B*; p≤0.01). Microscopic analysis of livers stained with H&E and Oil-Red-O revealed a significant reduction of liver TGs in clodronate-treated *ob/ob* mice compared with the PBS liposome-treated mice (Fig. 4*A*). Quantification of hepatic TG content revealed that KC depletion resulted in a 45% decrease in hepatic TGs in clodronate-treated *ob/ob* mice compared with the PBS liposome-treated controls (Fig. 4*C*; p≤0.05). Collectively, these data illustrate that clodronate liposome-mediated KC depletion, in two mouse models of obesity, results in a marked reduction of hepatic TG content and amelioration of obesity-induced hepatic steatosis. Notably, this reduction of hepatic TGs occurs independently of VATM depletion in the *ob/ob* mouse model, demonstrating the importance of KCs in the regulation of hepatic lipid metabolism.

### Clodronate liposome-mediated KC depletion decreases hepatic lipogenic gene expression in DIO and *ob/ob* mice

**Figure 5:**
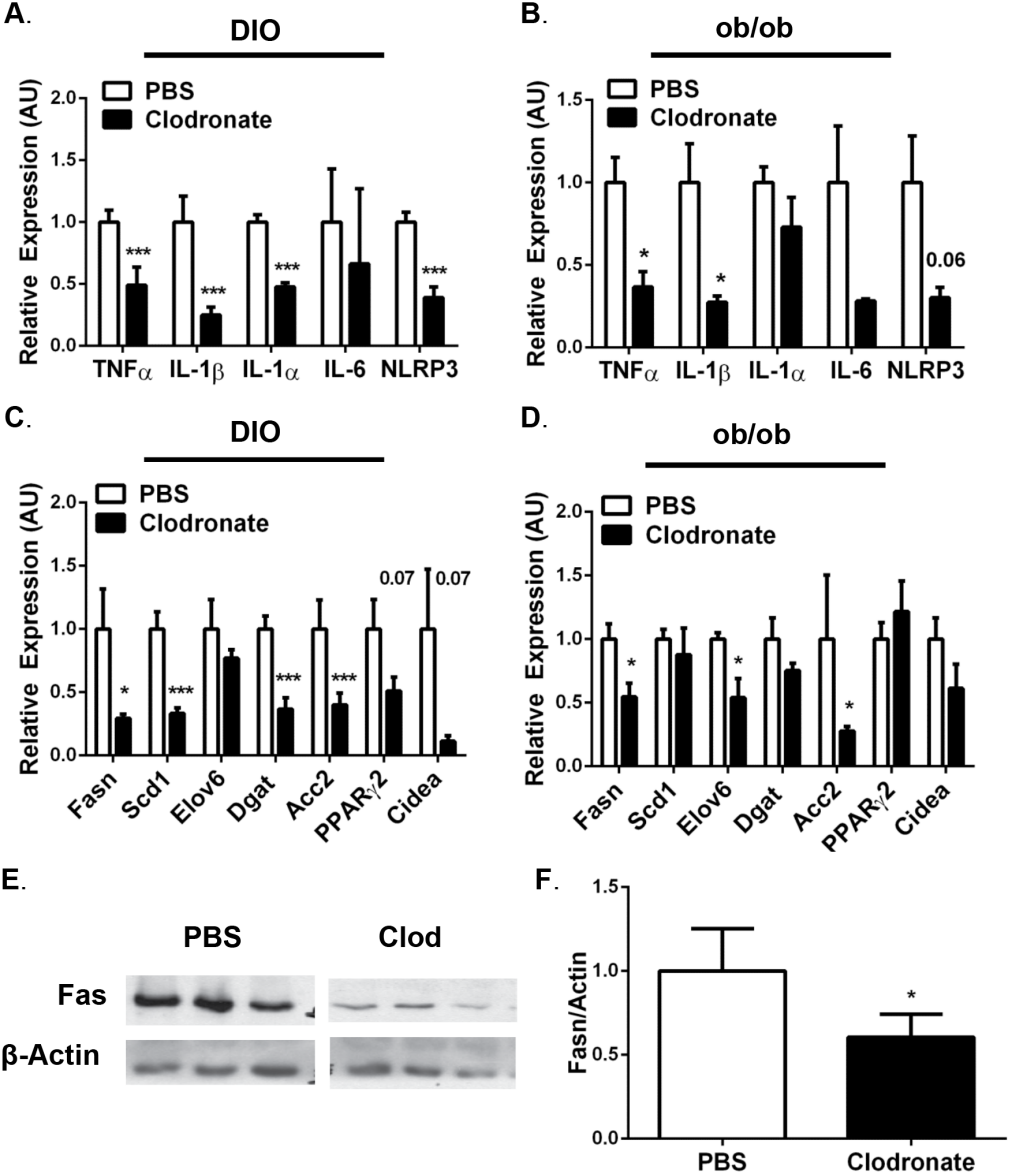
Clodronate liposome-mediated KC depletion in DIO and *ob/ob* mice significantly reduces hepatic expression of genes involved in inflammation and lipogenesis. Wild type mice fed a HFD for 13 weeks or 8-week *ob/ob* mice were injected with 2 doses of clodronate liposomes (250 mg/kg) or PBS liposomes by i.p. over 6 days with a 3-day interval. Data is representative of 12–16 DIO and 7–10 *ob/ob* animals. Liver was isolated, RNA was extracted and subjected to quantitative RT-PCR for inflammatory (TNF-α, IL-1β, IL-1α, IL-6, Nlrp3) and lipogenic (Fasn, Acc2, Dgat, Scd1, Elov6, PPARγ, Cidea) gene expression in *A,C.*) DIO and *B*,*D*) *ob/ob* mice. *E*.) Protein was extracted from the livers of DIO animals and Western blot analysis was performed to detect Fas expression. PBS and Clodronate samples are from the same film, cut due to sample separation. *F*.) Relative densitometry from *E*. Values represent the mean ± SEM. An unpaired student’s t*-*test was used for comparisons between groups. *P≤0.05, ***P≤0.001.

The striking reduction of hepatic TGs in the clodronate-treated mice could be mediated by different pathways, including increased fatty acid oxidation, increased very-low density lipoprotein (VLDL) secretion, reduced influx of non-esterified FFAs from AT, and/or reduced hepatic *de* *novo* lipogenesis [32]. To assess whether clodronate liposome treatment affected basal whole-body metabolic parameters, mice were fed a high fat diet (HFD) for 13 weeks and subjected to metabolic cage analysis throughout the duration of clodronate or PBS liposome treatment. We determined there were no differences in the food consumption, water intake or physical activity between groups throughout the course of liposome treatment (Table 1). There were also no differences in the energy expenditure, respiratory exchange ratio, or β-hydroxybutyrate levels in clodronate-treated DIO mice compared with PBS liposome-treated control mice (Table 1). Moreover, there were no changes in serum lipoprotein concentrations (Table 1) or expression of genes promoting fatty acid oxidation, including peroxisome proliferator-activated receptor-α (PPARα) or carnitine palmitoyltransferase-1a (Cpt1a) in the livers of clodronate-treated DIO mice compared with PBS liposome-treated mice (data not shown). These data suggest that whole body fatty acid oxidation is not affected by clodronate-treatment in DIO mice, and therefore we explored whether hepatic lipogenesis contributed to the reduced hepatic TG content in the clodronate-treated mice. Evaluation of liver samples by qRT-PCR analysis revealed reduced expression of peroxisome proliferator-activated receptor-γ (PPARγ) and downstream genes that encode lipogenic enzymes including stearoyl-coenzyme A desaturase 1 (scd1), diacylglycerol O-acyltransferase (dgat), fatty acid synthase (fasn), acetyl-CoA carboxylase-2 (acc2), as well as cell death-inducing DFFA-like effector A (cidea) that contributes to lipid droplet formation in both DIO and *ob/ob* clodronate-treated mice (Fig. 5*C*–*D*; p≤0.05). Consistent with these data, a 40% decrease in Fas protein expression was observed in the livers of the clodronate-treated DIO mice compared with PBS-liposome treated mice (Fig. 5*E*–*F*; p≤0.05). Collectively, these data suggest that depleting KCs in the livers of DIO and *ob/ob* mice is associated with decreased hepatic steatosis, which is mediated at least, in part, by down-regulating hepatic lipogenesis.

**Table 1:** Depletion of macrophages with clodronate-encapsulated liposomes improves fasting glycemia and insulin levels in DIO and *ob/ob* mice with no changes in the metabolic profile.

Metabolic Parameter	PBS (DIO)	Clodronate(DIO)	PBS(ob/ob)	Clodronate(ob/ob)
FBG (mg/dL)	235±11.9	**171±11.1	404±29.6	**256±20.0
Fasting Insulin (ng/mL)	2.0±0.3	*0.7±0.1	6.6±0.5	*3.9±1.0
FFAs (mmol/L)	0.30±0.02	*0.40±0.08	0.52±0.03	0.41±0.09
TGs (mg/dL)	56.6±17.8	61.5±13.9	32.2±5.1	**67.5±9.8
Cholesterol (mg/dL)	131±18.5	151±28.8	N.A.	N.A.
HDL (mg/dL)	121±11.3	138±19.1	N.A.	N.A.
LDL (mg/dL)	12±2.4	21±8.6	N.A.	N.A.
B-hydroxybutyrate (uM)	0.3±0.02	0.27±0.04	N.A.	N.A.
Food Intake (g/day)	2.67±0.17	2.51±0.21	N.A.	N.A.
Water Intake (g/day)	1.78±0.07	1.68±0.11	N.A.	N.A.
Total Activity (counts/day: 24 hr)	3290±381	3165±95	N.A.	N.A.
VO2 (ml/hr/kg: 24 hr)	4838±120	4759±78	N.A.	N.A.
VCO2 (ml/hr/kg: 24 hr)	3723±114	3650±61	N.A.	N.A.
EE (ml/hr/kg: 24 hr)	23.18±0.60	23.05±0.33	N.A.	N.A.
RER (ml/hr/kg: 24 hr)	0.771±0.01	0.759±.00	N.A.	N.A.

Wild type mice fed a HFD for 13 weeks or 8-week *ob/ob* mice were injected with 2 doses of clodronate liposomes (250 mg/kg) or PBS liposomes i.p. for 6 days with a 3-day interval. Data is representative of 12–16 DIO and 7–10 *ob/ob* animals. DIO mice were housed in metabolic cages prior to treatment for baseline analysis. Animals were housed in metabolic cages until completion of the experiment. EDTA plasma was drawn from the retro orbital sinus for serum analysis. Metabolic cage data is representative of 4–6 animals. Values represent the mean ± SEM. An unpaired student’s t*-*test was used for comparisons between groups. *p≤0.05, **≤0.01.

### Clodronate liposome-mediated KC depletion reduces hepatic inflammation in DIO and *ob/ob* mice

Inflammasome activation and the subsequent increase of IL-1β in obesity have received much attention because mouse models deficient in inflammasome components including NLR family pyrin domain containing 3 (Nlrp3), apoptosis-associated speck-like protein containing CARD (ASC), and caspase-1 implicate their involvement in the progression of obesity-induced hepatic steatosis [6], [11], [33], [34]. To explore the underlying mechanism by which macrophage depletion ameliorated hepatic steatosis, we analyzed inflammatory gene expression in the livers of clodronate liposome versus PBS liposome-treated DIO and *ob/ob* mice by qRT-PCR. Consistent with macrophage depletion, we observed a 40%–75% reduction in the mRNA expression of pro-inflammatory cytokines TNF-α, IL-1β, IL-1α and the Nlrp3 in the livers of clodronate-treated DIO mice (Fig. 5*A*; p≤0.001). We observed an even more robust 65%–72% reduction in the expression of these genes in the livers of clodronate liposome-treated *ob/ob* mice compared with PBS liposome-treated *ob/ob* mice (Fig. 5*B*; p≤0.05). However, no changes in the mRNA expression of IL-1α were observed in clodronate-treated *ob/ob* mouse livers (Fig. 5*B*). Taken together, these data demonstrate that clodronate-liposome mediated KC depletion decreases the inflammatory program in steatotic livers of obese mice.

### Physiological concentrations of recombinant IL-1β stimulate TG accumulation in isolated primary hepatocytes

**Figure 6:**
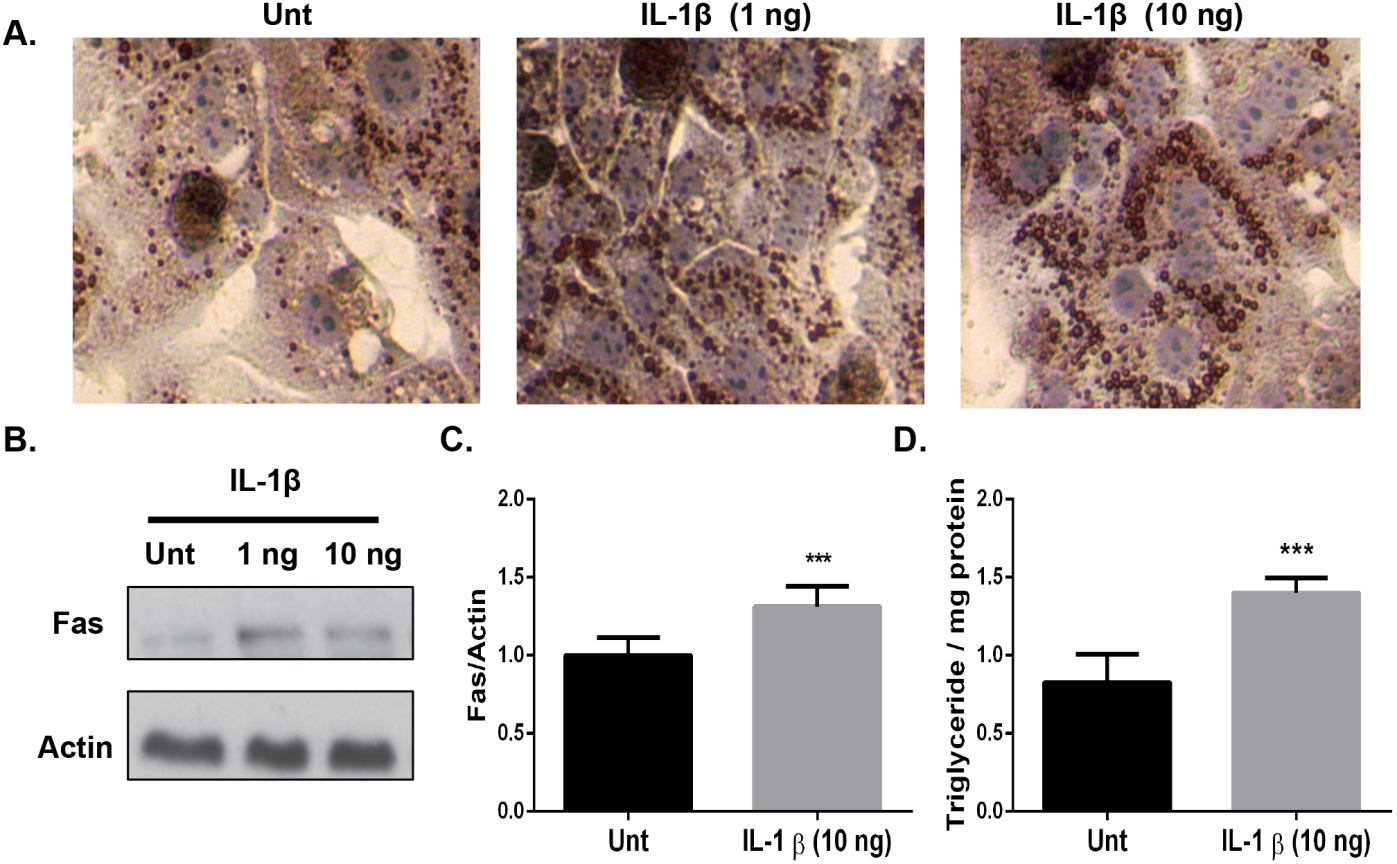
Physiological concentrations of IL-1β elicit a biological response in primary mouse hepatocytes to increase TG accumulation and lipogenic gene expression. Primary hepatocytes from wild type mice were isolated via perfusion, stimulated with the indicated doses of recombinant mouse IL-1β and evaluated after 24-hours. *A*.) Cells were fixed in 10% formalin and stained with Oil-Red-O to assess TG accumulation. *B*.) Fas expression was analyzed in primary hepatocyte cell lysates after a 24-hour stimulation with the indicated doses of recombinant IL-1β and normalized to β-actin. *C*). Relative densitometry from B. *D*.) TGs were extracted and measured after a 24-hour stimulation with recombinant IL-1β (10 ng/mL) and normalized to the amount of total protein. The data are representative of 4 experiments. All stimulations were performed in duplicate. Values represent the mean ± SEM. A paired student’s t*-*test was used for comparisons between groups *P≤0.05, ***P≤0.001.

Recent reports have implicated the involvement of the Nlrp3 inflammasome in the development of hepatic steatosis [6], [33], [35]. Based upon the reduced Nlrp3 and IL-1β mRNA expression in the livers of both the DIO and *ob/ob* clodronate-treated mice, we hypothesized that IL-1β might play a key role in steatosis development in hepatocytes. We isolated primary hepatocytes and assessed TG accumulation after 24-hour treatment with recombinant IL-1β or PBS (Fig. 6). Microscopic evaluation revealed a dose-dependent increase in TG accumulation with increasing physiological doses of IL-1β as measured by Oil-Red-O staining (Fig. 6*A*) [35]. To quantify the increased TG accumulation we measured total hepatocyte TGs and found a 50% increase in the recombinant IL-1β (10 ng/ml) treated hepatocytes compared with untreated cells (Fig. 6*D*; p≤0.001). Western blotting revealed a 30% increase in Fas protein expression in the recombinant IL-1β (10 ng/ml) treated hepatocytes compared with untreated cells (Fig. 6*B*–*C*; p≤0.05). Taken together, these data support the hypothesis that IL-1β promotes TG accumulation by upregulating *de* *novo* lipogenesis in primary hepatocytes and is important for the pathogenesis of obesity-induced steatosis.

### Pharmacological blockade of the IL-1 signaling pathway ameliorates diet-induced steatosis in mice

**Figure 7:**
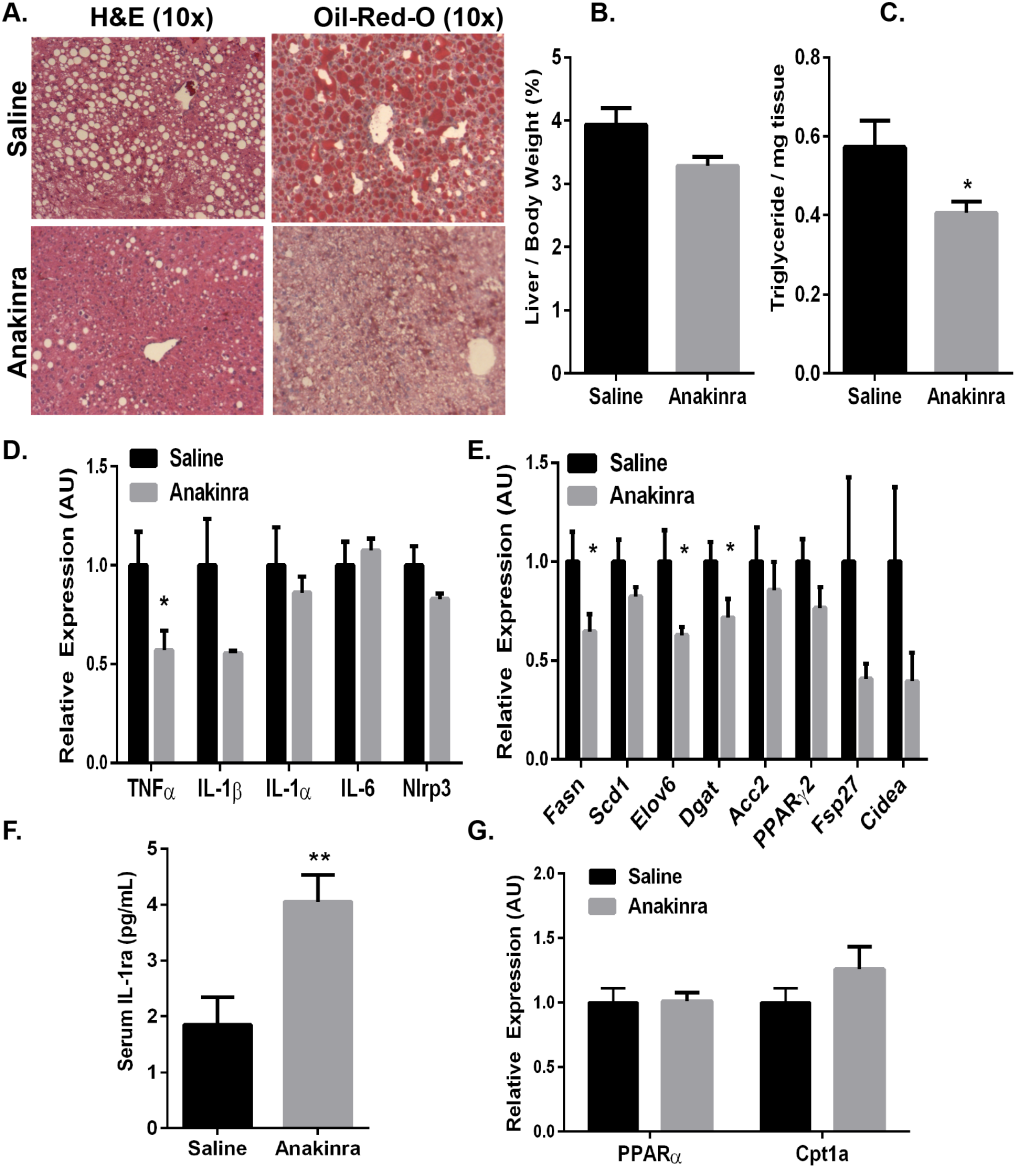
Pharmacological intervention via inhibition of IL-1 signaling ameliorates diet-induced steatosis in DIO mice. Wild type mice were fed a HFD for 13 weeks. At 32 days prior to sacrifice, mice were started on daily injections of IL-1Ra (Anakinra; 32 mg/kg) or saline by i.p administration. Data are representative of 10 mice per group. *A*.) Livers were isolated, fixed in 10% formalin, embedded in paraffin, and stained with H&E or frozen in OCT and stained with Oil-Red-O to assess steatosis. *B*.) Livers were isolated, weighed and represented as a percentage of total animal body weight. *C*.) Total TGs were extracted and normalized to tissue weight. RNA was extracted and subjected to quantitative RT-PCR for expression of genes involved in *D*.) inflammation (TNF-α, IL-1β, IL-1α, IL-6, Nlrp3), *E*.) lipogenesis (Fasn, Acc2, Dgat, Scd1, Elov6, PPARγ, Cidea) and *G*.) fatty acid oxidation (PPARα, Cpt1a). *F*.) EDTA plasma was drawn via the retro orbital sinus and serum was analyzed for IL-1Ra by ELISA. Values represent the mean ± SEM. An unpaired student’s t*-*test was used for comparisons between groups *P≤0.05, **P≤0.01.

**Figure 8:**
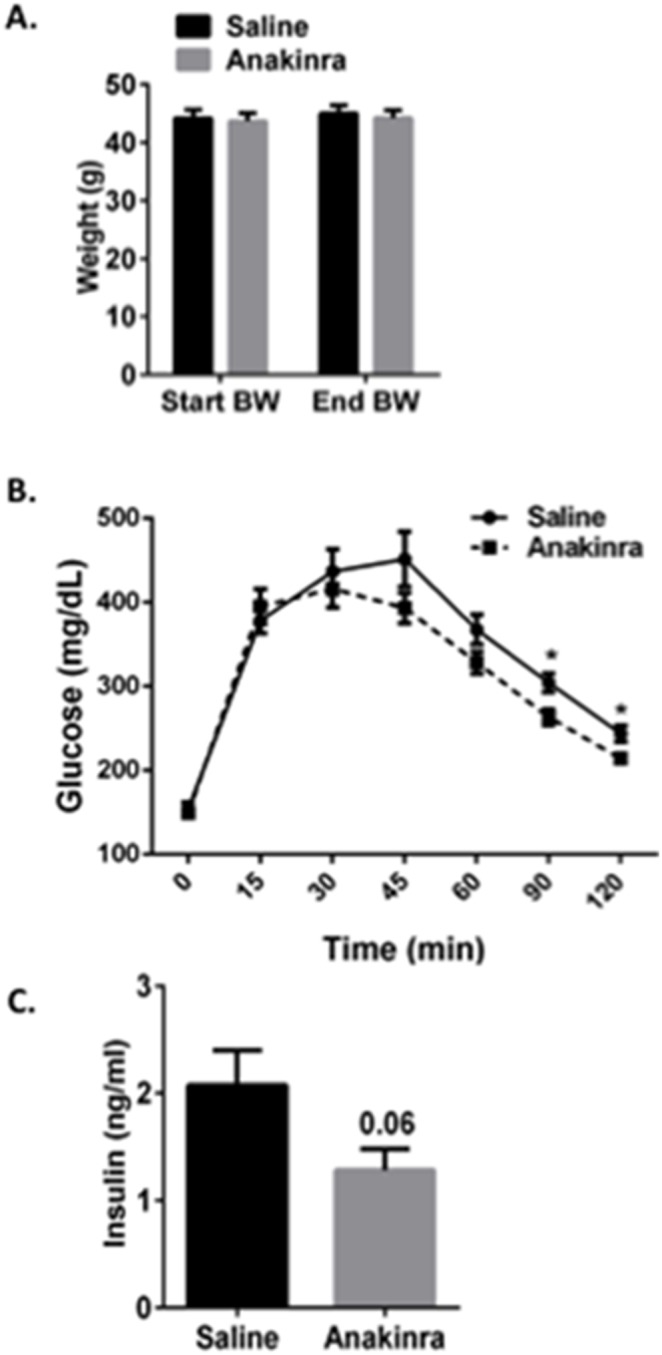
Pharmacological intervention via inhibition of IL-1 signaling improves the glucose tolerance in DIO mice. Wild type mice were fed a HFD for 13 weeks. At 32 days prior to sacrifice, mice were started on daily injections of IL-1Ra (Anakinra; 32 mg/kg) or saline by i.p. administration. Data are representative of 10 mice per group. At the end of 13 weeks, Anakinra-treated mice displayed no changes in *A.*) total animal body weight and were more glucose tolerant as demonstrated by a reduction in *B*.) area under the curve (AUC) for the intraperitoneal GTT and *C*.) fasting insulin levels. Values represent the mean ± SEM. An unpaired student’s t*-*test was used for comparisons between groups. *P≤0.05, **P≤0.01.

The activity and signaling cascades downstream of IL-1α and IL-1β are tightly regulated by IL-1Ra, an endogenous antagonist of the IL-1 receptor [36], [37], [38]. IL-1Ra negatively regulates IL-1 signaling by binding and blocking its receptor without activation, and mice lacking IL-1Ra has amplified steatosis compared with wild type animals [39]. To directly determine whether pharmacological inhibition of IL-1 signaling has a protective effect against NAFLD in DIO mice, we treated mice with recombinant human IL-1Ra (Anakinra). We fed mice a HFD for 9-weeks to establish IR and steatosis and then i.p. administered IL-1Ra (32.5 mg/kg) or an equal volume of saline daily for the final 32 days of a 13-week HFD. Serum recombinant human IL-1Ra levels were increased in IL-1Ra-treated mice compared with saline-treated mice, confirming that a physiological excess of IL-1Ra was present in circulation (Fig. 7*F*; p≤0.01). To confirm that IL-1Ra was biologically active in these mice, we performed intraperitoneal glucose tolerance tests (GTTs) 28 days after the start of injections (Fig. 8*B*). Consistent with previous studies [20], [21], IL-1Ra treatment improved glucose tolerance without affecting the total animal body weight in DIO mice (Fig. 8; p≤0.05).

Upon dissection of the mice, a macroscopic reduction in the steatotic appearance of the IL-1Ra-treated mouse livers was observed compared with saline-treated mice (Fig. 7*A*–*C*). In agreement with this observation, liver weights as a percentage of body weight were reduced by approximately 20% in IL-1Ra-treated mice compared with saline-treated mice (Fig. 7*B*). Microscopic analysis of steatosis, as assessed by H&E and Oil-Red-O staining, revealed a significant improvement in the liver steatosis in the IL-1Ra-treated animals compared with saline-treated controls (Fig. 7*A*). To quantify this improvement, we measured total hepatic TGs and found a 30% reduction in IL-1Ra-treated mice compared with saline-treated mice (Fig. 7*C*; p≤0.05). Importantly, these data demonstrate that inhibition of IL-1 signaling by IL-1Ra administration in obese mice is sufficient to reduce hepatic TG content and improve diet-induced hepatic steatosis.

To further confirm the activity of IL-1Ra in these mice, RNA was extracted from the livers of the IL-1Ra or saline-treated DIO mice to evaluate the mRNA expression of pro-inflammatory cytokines, including IL-1β and TNF-α. There was a reduction in liver TNF-α, IL-1β, however, we observed no changes in IL-1α, IL-6 or Nlrp3 expression in the IL-1Ra-treated mice compared with saline-treated control mice (Fig. 7*D*; p≤0.05). To assess whether the improved steatosis in the IL-1Ra-treated mice was due to altered lipid metabolism, genes involved in fatty acid synthesis and fatty acid oxidation were analyzed. Consistent with our previous findings in the clodronate-treated mice, reduced expression of genes involved in fatty acid synthesis and lipogenesis (elov6, dgat, fasn, acc2) was observed in the IL-1Ra-treated mice compared with saline-treated control animals (Fig. 7*E*; p≤0.05). We observed no changes in the expression of genes related to fatty acid oxidation, such as PPARα or Cpt1a (Fig. 7*G*). Taken together, these data demonstrate that IL-1Ra administration protects against hepatic steatosis to a similar extent as clodronate liposome-mediated KC depletion in obese mice. Importantly, these findings also suggest that IL-1 signaling plays a significant role in the progression of obesity-induced steatosis by activating the lipogenic pathway in hepatocytes.

## Discussion

The key results presented here demonstrate that the macrophage-derived inflammatory mediator IL-1β potentiates the hepatic manifestation of the metabolic syndrome by stimulating lipogenesis and hepatic steatosis in obese mice (Figs. 5–7). Utilizing clodronate liposomes to selectively deplete liver KCs in genetically obese *ob/ob* mice, we report remarkable amelioration of hepatic steatosis and significant reductions in hepatic inflammation and lipogenic gene expression (Figs. 4–5). Similar results were obtained in the DIO model treated with clodronate liposomes (Figs. 1–2 and 5). We also show that pharmacological blockade of IL-1 signaling by IL-1Ra dramatically improves hepatic steatosis by significantly decreasing inflammation and lipogenic gene expression in DIO mouse livers (Fig. 7). Taken together, these data indicate that inflammation not only drives liver disease progression, but also facilitates the increased lipogenic program and increased TG content in obese mouse livers.

Inflammation in both VAT and liver appear to play important roles in mediating disrupted metabolism in obesity. Immune activation and macrophage recruitment to obese AT is correlated with systemic IR and may contribute to adipocyte dysfunction with limited ability to sequester triglyceride and ectopic lipid deposition [40]. Lipid-laden KCs in the liver, resulting from increased influx of excess FFAs in circulation and increased hepatic *de* *novo* lipogenesis, are primed to recruit immune cells and exhibit a pro-inflammatory phenotype that exacerbates hepatic inflammation and TG accumulation, thus promoting obesity-induced steatosis and NAFLD [41]. However, the specific role of KCs in obesity-induced hepatic steatosis remains unclear because the authors of numerous studies on this point have come to contradictory conclusions [12], [22], [24], [28]. For example, Bu et al, administered clodronate liposomes by i.p. as a preventative treatment against DIO and reported protection against diet-induced steatosis; however, the data show robust decreases in VATM content, but only modest reductions in KC content in the livers of clodronate-treated DIO mice [28]. The use of CD11c-diptheria toxin transgenic mice to deplete macrophages in AT revealed normalization of insulin sensitivity in obese mice; however, this method also did not alter KCs in the liver [9]. Feng and colleagues reported significant improvements in obesity-induced steatosis with marked reductions in VATM and KC content in clodronate liposome-treated DIO mice [12]. On the contrary, Clemente, et al reported significant increases in both hepatic TG and VATM content in DIO mice treated with clodronate liposomes by i.p., and Lanthier, et al reported significant reductions in KCs without changes to the hepatic TG or VATM contents after intravenous clodronate liposome administration in DIO mice [22], [24]. Thus, the differences in methods used amongst these studies, including dissimilar administration routes, inconsistent injection timelines, varied diets and feeding schedules, and inconsistent clodronate concentrations have confounded our understanding of the role of KCs in hepatic lipid metabolism.

We approached this problem by studying both a DIO mouse model and a genetic mouse model of obesity. Our studies presented here on the *ob/ob* mouse, which responded to the clodronate treatment with selective KC depletion without macrophage cell depletion in VAT or SAT, was particularly insightful (Fig. 3). This selectivity of macrophage depletion of KCs but not VATMs in the *ob/ob* mouse may relate to an impaired ability of AT macrophages from *ob/ob* mice to take up liposomes. Li et al demonstrated that lipid-laden macrophages from the peritoneum and atherosclerotic lesions of *ob/ob* mice have an impaired ability to clear apoptotic cells, suggesting a generalized defect in the phagocytic ability of *ob/ob* macrophages [42]. Additionally, clodronate preferentially depletes F4/80^high^ CD11b^low^ macrophages, consistent with the expression profile of KCs, whereas clodronate does not effectively deplete F4/80^low^ CD11b^high^ macrophages from other tissues or in systemic circulation [43], [44]. For these reasons, by utilizing the *ob/ob* mouse, we were able to characterize the metabolic consequences of selective KC depletion in the absence of generalized macrophage depletion in other tissues. Thus, the striking improvement of obesity-induced hepatic steatosis in the clodronate-treated *ob/ob* mice reveals a profound regulation by KCs on hepatic lipid metabolism.

Our data also revealed remarkable improvements in obesity-induced hepatic steatosis with reductions in liver weight and hepatic TG content in the clodronate-treated DIO mice when compared with PBS liposome-treated control animals (Fig. 2). A recent report by Bu et al suggests that this clodronate liposome-induced reduction of hepatic TGs in DIO mice is dependent upon VATM depletion [28]. In our study, cell depletion due to clodronate liposomes was observed for both KC and VATMs in the DIO mouse model (Figs. 1–2), precluding a specific interpretation regarding which pool of cells may be responsible for the metabolic effects observed in this mouse model. However, our results in the *ob/ob* mouse showing a selective KC effect on hepatic lipids suggests that KCs may also drive liver TG accumulation in the DIO model, and that both KCs and VATMs are important in modulating obesity-induced hepatic steatosis.

The hallmark of NAFLD is the excessive accumulation of TG in hepatocytes caused by alterations in hepatic lipid metabolism. Mechanisms contributing to excessive hepatic TG accumulation in obesity are disruptions in lipid disposal via β-oxidation or VLDL secretion, but primarily due to enhanced hepatic *de* *novo* lipogenesis and increased delivery of circulating FFAs to the liver [45]. Increased lipolysis of expanded AT and the consequent rise in circulating FFAs account for 60% of the hepatic TGs in obese patients presenting with NAFLD [46]. Clodronate liposome-treatment reduced VATM content in DIO mice, and these mice present with increased circulating FFAs (Fig. 1*A*–*C*, Table 1). These data are consistent with Kosteli, et al. demonstrating that macrophage cell depletion in VAT leads to increased expression of the TG lipase ATGL and increased lipolysis in mice [47]. We report no changes in circulating FFAs in clodronate-treated *ob/ob* mice, and deem this to be due to the inability to deplete VATMs in this model; however, these mice exhibit increased circulating TGs (Fig. 3*A*–*C*, Table 1). Importantly, the rise in circulating FFAs in clodronate-treated DIO mice is accompanied by remarkable hepatic TG clearance without changes in the cholesterol profile, circulating ketones, or energy expenditure when the mice are subjected to metabolic cage analysis (Fig. 2, Table 1). Using a multiple-stable-isotope approach, Donnelly et al. estimated *de* *novo* lipid synthesis accounts for 30% of the hepatic TG content in NAFLD patients [32]. We report reduced expression of genes related to *de* *novo* hepatic lipogenesis (Fasn, Acc2, Dgat, Scd1) in both DIO and *ob/ob* clodronate-treated mice (Fig. 5*C*–*F*). Consistent with our data, the liver-specific Scd1 knockout (KO) mice are protected from obesity-induced hepatic steatosis, while exhibiting reduced rates of hepatic fatty acid synthesis and decreased expression of key lipogenic enzymes (Fasn and Acc1/2) [48]. Mao, et al generated liver-specific Acc1 KO mice with decreased rates of *de* *novo* lipogenesis, however a compensatory up regulation of Acc2 occurred in other studies utilizing these mice [49], [50]. Inhibition of Acc1/Acc2 by antisense oligonucleotides reversed diet-induced hepatic steatosis in mice [49], [50], [51]. Collectively, our data suggests that the clearance of hepatic TGs after clodronate liposome-mediated KC depletion in the livers of obese mice is associated with decreased hepatic steatosis which is mediated, at least in part, by down regulating hepatic *de* *novo* lipogenesis.

Obese humans that present with NAFLD have increased circulating and hepatic levels of TNF-α, IL-1β, IL-6 and other acute phase proteins when compared with lean control subjects [7], [18], [52]. KC depletion significantly reduced hepatic gene expression of these pro-inflammatory cytokines in both clodronate-treated DIO and *ob/ob* mice (Fig. 5*A*–*B*). We also report decreased Nlrp3 expression, an essential inflammasome component that is necessary for caspase-1 activation and IL-1β release, in both clodronate-treated DIO and *ob/ob* mice compared with PBS liposome-treated controls (Fig. 5*A*–*B*). Our data show no changes in the gene expression of inflammasome-independent cytokine, IL-1α, in clodronate-treated *ob/ob* mouse livers (Fig. 5*B*). Even though clodronate-mediated KC depletion improves hepatic steatosis in both models, we only observe a decrease in IL-1α expression in clodronate-treated DIO mouse livers compared with control mice (Fig. 5*A*). Adoptive transfer experiments reveal that IL-1α is a hepatocyte-derived cytokine, rather than from KCs or recruited bone marrow-derived cells in obese mice [53], [54]. Furthermore, IL-1α and IL-1β are expressed at different phases of the inflammatory response and are derived from different cell types, suggesting that they may have distinct biological roles [55]. Additionally, IL-1β KO mice are protected from diet-induced steatosis, whereas IL-1α KO mice develop steatosis [53]. Although in our studies we cannot discount IL-1α involvement, we hypothesize that it does not play a role in obesity-induced hepatic steatosis development. Thus, the decreases in hepatic inflammation after clodronate liposome-mediated KC depletion suggest that inflammasome activation required to increase IL-1β production from KCs is important for obesity-induced hepatic inflammation.

IL-1β rapidly increases hepatic lipid accumulation *in* *vivo* by acutely increasing the rates of hepatic fatty acid synthesis [34], [56]. To further address the steatogenic potential of IL-1β, we assessed whether IL-1β would increase hepatic TG accumulation in a cell autonomous manner. IL-1β treatment increased TG accumulation and expression of the key lipogenic enzyme, Fas, in primary hepatocytes (Fig. 6). In agreement with the decreased hepatic inflammation observed in clodronate-treated mice, caspase-1-deficient mice (that lack IL-1β expression) exhibit attenuated diet-induced hepatic steatosis and significant decreases in hepatic lipogenic gene expression compared with wild type control mice [33]. These data suggest that IL-1β influences hepatocyte lipid metabolism in obese mice by stimulating the *de* *novo* lipogenesis pathway to drive hepatic TG accumulation. However, Stienstra and colleagues suggested that IL-1β-mediated suppression of hepatic PPARα and fatty acid oxidation was responsible for hepatic lipid accumulation in obesity [57]. We report no changes in the respiratory exchange ratio, oxygen consumption or circulating β-hydroxybutyrate levels in clodronate-treated DIO mice compared with PBS liposome-treated mice, suggesting functional hepatic fatty acid oxidation in our clodronate-treated DIO mice (Table 1).

The inflammatory response associated with obesity arises from an activation of the innate immune system, concurrently increasing anti-inflammatory cytokines to neutralize these pro-inflammatory changes. IL-1Ra, a naturally occurring antagonist of IL-1 signaling, is released as an acute phase protein and is produced by hepatocytes, macrophages/monocytes, and even adipocytes to balance the inflammatory effects of IL-1α/β [39], [58], [59]. Circulating IL-1Ra positively correlates with obesity, and IL-1Ra KO mice exhibit a dramatic exacerbation of hepatic steatosis [39], [60]. Pioglitazone, an oral anti-diabetic drug, decreases hepatic steatosis and attenuates the subclinical inflammation in NAFLD patients; however prominent side effects include increased adiposity and weight gain [61], [62]. Anakinra, also used as a therapeutic in obese, diabetic patients, improves pancreatic islet function and glucose tolerance, but has not been studied in the context of NAFLD [18]. We demonstrated here that pharmacological blockade of IL-1 signaling by IL-1Ra administration is sufficient to improve hepatic steatosis and the metabolic profile in DIO mice (Figs. 7–8). Daily IL-1Ra administration to DIO mice significantly improved glucose tolerance as detected by GTT without changes to the total animal body weight (Fig. 8*A*–*D*). Collectively, we report a significant improvement in obesity-induced steatosis as assessed by hepatic Oil-Red-O staining, decreased liver weight and reductions in the hepatic TG content of IL-1Ra-treated mice compared with saline-treated control mice (Fig. 7*A*–*C*). The expression of pro-inflammatory cytokines and genes involved in fatty acid synthesis (Fasn, Dgat, Scd1) are reduced significantly in the livers of IL-1Ra-treated mice, thus confirming our findings in the clodronate-treated DIO and *ob/ob* mouse models (Fig. 5 and Fig. 7*D*–*E*). Importantly, inhibition of IL-1 signaling did not affect fatty acid oxidation gene expression (PPARα and Cpt1a) (Fig. 7*G*). These data suggest that inhibition of IL-1 signaling by IL-1Ra administration is sufficient to improve obesity-induced hepatic steatosis, in part, by decreasing hepatic lipogenic gene expression and TG accumulation in DIO mouse livers.

In summary, our findings demonstrate that KC depletion in two mouse models of obesity markedly reduces hepatic inflammation and obesity-induced steatosis. With selective depletion of KCs in the *ob/ob* mouse, our data suggests that this improvement in hepatic steatosis is independent of VATM depletion, and that KCs and KC-derived cytokines, including IL-1β are important for hepatic metabolism regulation. Our data also suggests that the significant reduction in hepatic TGs observed in both clodronate-treated DIO and *ob/ob* mice is mediated, in part, by down regulating hepatic inflammation and *de* *novo* lipogenesis. Additionally, we report that inhibition of IL-1 signaling by administration of IL-1Ra markedly improves hepatic steatosis in DIO mice by significantly reducing hepatic inflammation and lipogenic gene expression. Therefore, the results herein suggest that IL-1β signaling mediates hepatocyte TG accumulation by driving the *de* *novo* lipogenic signaling pathway in obese mouse livers and that IL-1β represents a promising therapeutic target in the treatment of obesity-induced NAFLD.
